# Targeted Therapy in Ovarian Cancer

**DOI:** 10.1155/2010/740472

**Published:** 2010-01-14

**Authors:** Lyndsay J. Willmott, John P. Fruehauf

**Affiliations:** Chao Family Comprehensive Cancer Center, University of California Irvine, Orange, CA 92868, USA

## Abstract

Ovarian cancer is the most common cause of mortality of tumors from gynecologic origin and is often diagnosed after patients have already progressed to advanced disease stage. The current standard of care for treatment of ovarian cancer includes cytoreductive surgery followed by adjuvant chemotherapy. Unfortunately, many patients will recur and ultimately die from their disease. Targeted therapies have been evaluated in ovarian cancer as a method to overcome resistant disease. Angiogenesis inhibitors have shown success in many tumor types and have also demonstrated promise in trials involving patients with ovarian cancer. PARP inhibitors may be potentially active agents in patients with BRCA-associated ovarian cancer. Trials that have evaluated combinations of targeted agents have often revealed untoward toxicities, thus tempering enthusiasm for this approach.

## 1. Introduction

Ovarian cancer is the most common cause of mortality from gynecologic cancer and will be responsible for 14 600 cancer related deaths this year. Secondary to vague presenting symptoms and the lack of effective screening, most patients will present with advanced disease. The current standard of care for ovarian cancer therapy is surgery followed by adjuvant carboplatin and taxane-based chemotherapy. Unfortunately, these protocols often do not allow for cure at initial diagnosis, and many patients will often recur and eventually die from their disease. Chemoresistance is an important hurdle in the treatment of recurrent cancer. Targeted therapy has subsequently come to the forefront of research and clinical trials in an effort to overcome resistant disease and achieve improvement in patient outcomes.

## 2. Epidemiology

Ovarian cancer is the second most common gynecologic malignancy, but is the most common cause of mortality from gynecologic cancer. It accounts for about 3 percent of all cancers among women and is the fifth most common cause of cancer-related death in women [1]. Approximately 21 550 cases will be diagnosed and 14 600 deaths will occur this year [2].

Surveillance Epidemiology and End Results (SEER) database shows that the incidence of ovarian cancer has decreased over the past 30 years [2]. Age-based incidence increases from 0.26/100 000 at age 5–9 to a peak of 58.3/100 000 at age 80–84. Following this, there is a downward trend in incidence rate. The lifetime risk of ovarian cancer in the general population is 1.7 percent. Most women who are diagnosed with epithelial ovarian cancer (EOC) are between the ages of 40 and 65.

## 3. Diagnosis and Initial Treatment

Unfortunately, the initial signs and symptoms of ovarian cancer are vague. These can include nonspecific complaints of bloating, gastrointestinal symptoms, and pain [3]. The subtle nature of symptoms can often delay patient presentation. It is important for a provider to retain a high index of suspicion if a patient presents with abdominal or pelvic symptoms, particularly if these symptoms occur daily, are more severe than expected, or present as a constellation of complaints. Secondary to lack of screening tools and the indolent nature of presenting symptoms, ovarian cancer often presents when patients have already progressed to disseminated disease. A prior analysis by the International Federation of Gynecology and Obstetrics showed that distribution by stage is I (23 to 33 percent), II (9 to 13 percent), III (46 to 47 percent), and IV (12 to 16 percent) [4]. Those who present with advanced stage are often incurable.

Cytoreduction is the goal in initial surgical therapy for patients with ovarian cancer. Decreasing the remaining tumor burden has been shown to improve response to postoperative systemic chemotherapy. This finding is biologically plausible, in that small tumors are better perfused and more mitotically active, thereby allowing chemotherapeutic drugs to have better efficacy. A meta-analysis of over 53 studies with advanced stage ovarian carcinoma treated with platinum-based chemotherapy found a 5.5 percent increase in median survival for every 10 percent increase in the proportion of patients achieving maximal cytoreduction, which was defined as less than or equal to 3 cm in the analysis [5].

The current standard of care for initial adjuvant chemotherapy in EOC is a platinum drug, usually carboplatin, and a taxane. The Gynecologic Oncology Group (GOG) evaluated the efficacy of cisplatin versus carboplatin in a noninferiority trial. The authors concluded that a chemotherapeutic regimen consisting of carboplatin plus paclitaxel results in less toxicity, is easier to administer, and is not inferior, when compared with cisplatin plus paclitaxel [6].

## 4. Second Line and Targeted Therapy

Unfortunately, despite optimal cytoreduction and adequate adjuvant therapy, many patients with EOC will experience disease recurrence. Over 70–80 percent of patients will relapse and ultimately die of their disease [7]. Therapy for recurrent disease is varied and depends upon time to recurrence.

Patients are categorized into groups based on their disease-free period, including platinum-sensitive (those patients who recur greater than 12 months after therapy), partially platinum-sensitive (those who recur between 6–12 months after therapy), platinum-resistant (those who recur before 6 months after therapy), and platinum-refractory (those who never achieve disease free status). Traditionally, patients who recur more than 6 months after initial therapy are given a second course of platinum-taxane-based chemotherapy. Platinum-sensitive disease has a greater than 50 percent response rate to single agent carboplatin, while resistant disease has a 10–20 percent response rate and refractory disease response is even lower [8]. The latter groups are therefore typically treated with other FDA approved chemotherapy regimens including pegylated liposomal doxorubicin, gemcitabine, topotecan, and etoposide [9].

The bane of ovarian cancer therapy is the failure of currently established treatment protocols to allow for cure of the disease at diagnosis, even in patients with initially chemosensitive tumors. Despite efforts of clinical trials to identify more efficacious regimens to overcome the chemoresistance encountered after front-line platinum-taxane treatment, clinical response to second-line therapy continues to be short lived and results in only marginal improvements in progression free and overall survival [8]. In response to this challenge, the idea of overcoming resistant disease with targeted therapy has come to the forefront of investigation in ovarian cancer therapy.

## 5. Angiogenesis Targeted Therapy

Angiogenesis is the development of new blood vessels in areas of new tissue growth. This is a normal phenomenon associated with routine processes including wound healing and embryogenesis. It is also an important process that occurs almost universally in solid tumors as a response to the expansion of the cancer mass and its subsequent growth away from existing blood supply. This causes the oxygen tension to decrease beneath physiologic levels needed for oxidative metabolism [10].

An important interplay of proangiogenic signaling occurs in response to the hypoxic state. A protein called hypoxia-inducible factor (HIF) 1 alpha is stabilized in these conditions and enters the nucleus where it forms a complex with another protein (HIF 1 beta) [11]. This complex is then able to act as a transcription factor allowing upregulation of growth factors including vascular endothelial growth factor (VEGF) [12, 13]. The VEGF family includes six closely related molecules, but the most important angiogenic agent is VEGF-A. 

Molecular markers of angiogenesis have been studied in ovarian cancer. Prior studies have shown associations between VEGF-A levels and microvessel density in primary tumors and disease extent as well as progression-free and overall survival following initial antiangiogenic therapy [14]. Preclinical models have also shown the importance of the VEGF pathway in ascites formation [15, 16].

Bevacizumab is a monoclonal antibody directed against VEGF-A. Studies evaluating this agent have shown improved survival in colorectal [17], breast [18], and lung cancers [19]. A GOG phase II study of bevacizumab in persistent or recurrent EOC or primary peritoneal carcinoma was performed by Burger et al. [20]. This study revealed a 21% clinical response rate. Of the 62 patients on trial, 25 experienced at least 6-months progression free survival (PFS), with a median PFS of 4.7 months and median overall survival of 17 months. This study was unique in that none of the patients experienced gastrointestinal perforation, a known complication of bevacizumab in other clinical trials. Cannistra et al. performed a phase II trial of single agent bevacizumab in patients with platinum-resistant disease [21]. As opposed to the GOG trial, this study was closed early secondary to the proportion of patients that experienced GI perforations (5/44), but the study did show a 16% response rate and a median durable response of 12 weeks. Toxic events that were similar between these two trials include hypertension and vascular thrombosis. Garcia and colleagues performed a phase II trial of bevacizumab that evaluated the use of bevacizumab and low-dose metronomic oral cyclophosphamide in recurrent ovarian cancer [22]. The authors found a 28% response rate with 6 month PFS of 28%; see Table 1. 

Based on the activity of bevacizumab as documented in these phase II trials, there are currently two trials that are ongoing to evaluate the activity of bevacizumab in the setting of front line adjuvant therapy. The first is GOG 218, a study that evaluates stages III and IV EOC patients who have undergone surgery and are subsequently randomized to one of three arms; arm 1 utilizes the traditional chemotherapy regimen of carboplatin (AUC 6) and paclitaxel (175 mg/m2) and placebo, arm 2 includes the active drugs of arm 1 and adds bevacizumab (15 mg/kg every 21 days for 6 cycles, starting with cycle 2), while arm 3 includes the drugs of arm 2 and adds maintenance bevacizumab given every 21 days to complete 22 cycles. A second trial is run by the Gynecologic Cancer InterGroup in Europe (ICON7) and is an open label trial. The ICON7 study population includes both high risk early stage disease (stage I-IIa with grade 3 or clear cell histology) and advanced disease IIb-IV EOC or primary peritoneal cancer. Patients are randomized to one of two arms: carboplatin and paclitaxel or carboplatin, paclitaxel, and bevacizumab. The bevacizumab arm also includes a maintenance schedule continuing the drug every three weeks for 12 cycles. The study aims to evaluate PFS as a primary endpoint and overall survival, duration of response, and response rate as secondary endpoints [23].

Bevacizumab has also been studied in conjunction with other targeted agents. A phase I study of bevacizumab and a vascular disrupting agent (VDA) combretastatin 4A phosphate (CA4P) in patients with advanced solid tumors demonstrated no additive toxicity and the evidence for efficacy was encouraging [24]. This is of interest because preclinical evidence exists for synergy between VDA, which causes a surge in VEGF-stimulated circulating endothelial progenitor cells, and bevacizumab, which suppresses this induced effect [25].

VEGF Trap is a fusion protein consisting of the extracellular domains of human VEGF-1 and -2. This protein binds to VEGF-A and placental growth factor. In mouse models VEGF Trap treatment resulted in decreased ovarian cancer growth and ascites [26]. Tew and colleagues reported on a phase II study evaluating patients with recurrent, platinum-resistant EOC. The participants received VEGF trap (2 or 4 mg/kg) administered intravenously every two weeks. This study yielded an 11% partial response, with grade 3/4 toxicities including hypertension, proteinuria, encephalopathy, and renal failure [27]. A phase II trial involving VEGF trap combined with docetaxel in patients with recurrent EOC, primary peritoneal cancer or fallopian tube cancer with measurable disease is currently ongoing [28]. Patients in this study will receive VEGF trap at the maximum tolerated dose (as determined in Phase I of the trial which has closed to accrual) over 1 hour on day 1 of course 1, followed by VEGF trap IV over 1 hour and docetaxel over 1 hour on day 1 in all subsequent courses. The courses repeat every 21 days in absence of disease progression or unacceptable toxicity. 

C-kit is a growth factor receptor of the tyrosine kinase subclass III family, the ligand of which is Stem Cell Factor, and is normally expressed in many cell lines, including gametocytes [29]. C-kit signaling promotes cell proliferation, differentiation, migration, adhesion, and survival [30]. The platelet derived growth factor receptor beta (PDGRF-B) gene encodes a cell surface tyrosine kinase receptor for members of the platelet derived growth factor family. This receptor is essential for cell migration and development of microvasculature.

Cediranib is an oral VEGFR-1, -2, and -3, PDGFR-B, and c-kit inhibitor. Hirte et al. performed a phase II trial of cediranib in patients with recurrent or persistent EOC, primary peritoneal or fallopian tube cancers [31]. The trial design initially included daily oral dosing of 45 mg, which was decreased to 30 mg continuously secondary to toxicity. Of the patients with platinum sensitive disease, 41% responded to therapy, while those with platinum-resistant disease demonstrated a 29% response rate. Significant side effects included diarrhea, hypertension, fatigue, and anorexia. Median time to progression was 4.1 months, while median overall survival was 11.9 months. A phase III study of cediranib in patients with platinum sensitive recurrent EOC is currently ongoing in Europe; see Table 2.

## 6. Epidermal Growth Factor Receptor

Epidermal growth factor receptor (EGFR) is overexpressed in 70% of cancers and is associated with chemoresistance, poor prognosis, and advanced disease at presentation [32, 33]. The mechanism of growth factor receptors is via activation of the intracellular tyrosine kinase domain, which triggers downstream targets and subsequently cell proliferation and survival [34]. Preclinical studies suggested that inhibiting this target might reverse chemoresistance and demonstrate antitumor activity [35–37]. Unfortunately, clinical trials evaluating drugs affecting these pathways, such as studies of EGFR tyrosine kinase inhibitors (gefitinib and erlotinib) and monoclonal antibodies directed against EGFR (cetuximab, panitumumab, and matuzumab), have not been met with significant success, showing only modest efficacy [8]. 

Gefitinib is a small molecule tyrosine kinase inhibitor that binds to the ATP-binding site of the EGF receptor and thereby prevents its activation. A GOG phase II study of gefitinib in patients with relapsed or persistent ovarian or primary peritoneal carcinoma assessed the activity and tolerability of a daily oral dose of 500 mg. The trial showed that only four of 27 eligible and evaluable patients exhibited progression-free survival greater than 6 months. One objective response was seen, and interestingly this patient was found to have the rare presence of an EGFR mutation. EGFR expression was associated with longer PFS (*P* = .008) and possibly longer survival (*P* = .082). Gefitinib was well tolerated, with dermatologic (15%) and diarrhea (30%) the most common grade 3 toxicities [38]. A phase II trial performed by the AGO Ovarian Cancer Study group evaluated gefitinib (500 mg/day) in combination with tamoxifen (40 mg/day) given until progression or unacceptable toxicity in patients with platinum-resistant EOC [39]. While this study demonstrated no tumor responses, 16 of 56 patients had stable disease. Notably, there was an 11% discontinuation rate secondary to side effects including diarrhea and skin rash.

Erlotinib is an oral epidermal growth factor receptor (HER1/EGFR) tyrosine kinase inhibitor. Gordon and colleagues performed a phase II study in patients with refractory, recurrent, HER1/EGFR positive EOC [34]. Patients received 150 mg erlotinib orally once a day for up to 48 weeks or until disease progression or dose-limiting toxicity. This study found little clinical activity, with an objective response rate of 6% (2/34), both of which were partial responses. Stable disease was seen in 15/34 patients. Rash (68%) and diarrhea (38%) were the most frequent adverse events. Erlotinib was recently investigated as a single agent medication in maintenance therapy after first-line chemotherapy in a large study performed by the European Organization for Research and Treatment of Cancer (EORTC), with results forthcoming. 

Disappointment was also encountered in clinical trials examining erlotinib in combination with other agents, where toxicity led to premature termination [40]. A phase II trial by Nimeiri and colleagues of bevacizumab (15 mg/kg) administered intravenously every 21 days and erlotinib (150 mg) given orally every day was performed in 13 patients with recurrent ovarian, primary peritoneal and fallopian tube cancer. This study showed a 15% response rate and seven patients had a best response of stable disease. Two patients had fatal gastrointestinal perforations, which lead to the early termination of the trial. 

Overexpression of ERBB2 is also found in patients with ovarian cancer. Trastuzumab, or Herceptin, is a monoclonal antibody directed against ERBB2, and has been studied in a phase II trial by the GOG. This study evaluated the drug in patients with recurrent or refractory ovarian or primary peritoneal carcinoma with overexpression of HER2 [41]. Patients initially received trastuzumab at a dose of 4 mg/kg, then weekly at 2 mg/kg. Patients without progressive or excessive toxicity could continue indefinitely, and those with stable or responding disease at 8 weeks were offered treatment at a higher dose (4 mg/kg) at time of progression. The authors reported that only 7% of the patients responded to treatment and a median time to progression of 2 months was seen. 

Pertuzumab is a monoclonal antibody that inhibits dimerization of ERBB2 with EGFR, ERBB3, and ERBB4. A phase II trial of single agent pertuzumab administered as an intravenous loading dose of 840 mg followed by 420 mg every three weeks (in cohort 1) and as 1050 mg every three weeks (in cohort 2) was performed in advanced, refractory ovarian cancer. The authors reported a 4.3% partial response rate and 6.8% of patients with stable disease lasting at least 6 months. Median PFS was 6.6 weeks [42]. Patients who were phosphoHER2 positive had a trend toward higher median PFS (20.9 weeks) versus those who were negative (5.8 weeks, *P* = .14). Two trials have evaluated the efficacy of pertuzumab when combined with chemotherapy, one phase II study in combination with carboplatin and another phase II trial in combination with gemcitabine [43, 44]. The gemcitabine and pertuzumab trial was performed in patients with platinum resistant EOC, and there was the suggestion of some benefit of pertuzumab in patients with low levels of ERBB3 mRNA expression and platinum-resistant disease; see Table 3.

## 7. Multikinase Inhibitors

Sorafenib is an oral multikinase inhibitor that targets the mitogen-activated protein kinase (MAPK) pathway or Raf/MEK/ERK pathway [45]. This drug also inhibits VEGFR-1, -2, and -3 and platelet-derived growth factor receptor (PDGFR) beta tyrosine kinase activity. Sorafenib is currently FDA-approved for treatment of advanced renal cell cancer, and the biologic rationale for attempting its use in other solid tumors is the fact that MAPK pathway is well conserved evolutionarily and may serve as a central and common target [23].

A phase II trial of single agent sorafenib in persistent or recurrent EOC or primary peritoneal cancer was performed by the GOG [46]. Patients received sorafenib 400 mg orally twice daily until disease progression or prohibitive toxicity. Of the 59 patients with measurable disease, there were 2 partial responders and 20 patients with stable disease, 30 patients had progressive disease reported, and 7 were unable to have their tumor assessed. Grade 3 and 4 toxicities included rash, gastrointestinal, cardiovascular, metabolic, and pulmonary.

Sorafenib has also been studied in conjunction with other medications. A Phase I dose escalation study of sorafenib (200 mg orally twice daily) and bevacizumab (5 mg/kg or 10 mg/kg intravenously every two weeks) showed six Response Evaluation Criteria in Solid Tumors (RECIST) partial responses in 13 ovarian cancer patients, with duration of response from 4 to over 22 months [47]. Unfortunately, this combination yielded significant toxicity, with grade 3 hypertension, diarrhea, hand-foot syndrome, thrombocytopenia, proteinuria, and two episodes of fistula formation at sites of disease response. A phase II trial evaluated sorafenib in combination with gemcitabine [48]. Patients were given gemcitabine 1000 mg/m^2^ intravenously weekly for 7 out of 8 weeks of the first cycle, then weekly for the first 3 weeks of a 4-week cycle and sorafenib 400 mg orally twice daily continuously. Using RECIST criteria, the authors reported 1 out of 18 evaluable patients had a partial response and 5 had a confirmed partial response by CA125 criteria. An additional 10 patients exhibited stable disease. Median time to progression was 5.4 months and overall survival was 13.3 months. The most frequent grade 3 and 4 toxicities were hematologic (lymphopenia, neutropenia, and thrombocytopenia), fatigue, hypokalemia, and hand-foot syndrome.

Imatinib mesylate inhibits abl, c-kit, and PDGFR tyrosine kinases, thereby inhibiting tumor growth. It is FDA approved for some forms of adult and child chronic myelogenous leukemia as well as gastrointestinal stromal tumors (GISTs). Activating mutations of kit have not been found in ovarian cancers, but abnormal kit expression has been described [49]. The activity of single agent imatinib in patients with recurrent EOC has been poor. A phase II trial of imatinib administered orally at 600 mg daily for six weeks and repeated in absence of measurable progressions was performed in patients with platinum and taxane-resistant ovarian and primary peritoneal cancer. This trial showed no complete or partial responders during a median followup of 6.6 months [50]. A phase II trial of imatinib mesylate (400 mg orally) in recurrent ovarian cancer with positive c-kit or PDGFR found no objective responders and a median PFS of only 2 months [51]. The GOG also conducted a phase II trial of single agent imatinib (400 mg orally twice daily) in recurrent or persistent EOC or primary peritoneal cancer [49]. Eligibility for this trial included expression of at least one target (c-kit, PDGFR-alpha, PDGFR-beta) in the tumor. Only 9/56 patients were progression free for at least 6 months, with a median PFS of 2 months and median overall survival of 16 months. The most common grade 3 and 4 toxicities included GI, pain, electrolyte disturbances, dermatologic, and neutropenia; see Table 4.

## 8. PARP Inhibition

**Figure 1:**
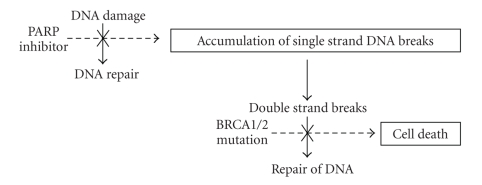
Poly(ADP-ribose) polymerase is involved in base excision repair of DNA single-strand breaks. If PARP is inhibited, these breaks can accumulate, potentially leading to double-strand breaks. These double-strand breaks are normally repaired by error-free homologous recombination, of which BRCA1 and BRCA2 proteins are involved. If these proteins are affected by somatic mutation or epigenetic silencing, eventual chromosomal instability and cell death can be seen.

Poly(ADP-ribose) polymerase (PARP) is an enzyme involved in repair of DNA single-strand breaks using the base excision repair pathway [52, 53]. A recent review by Yap et al. detailed the mechanism by which PARP inhibition can lead to cancer cell death. Inhibition of PARP leads to the accumulation of DNA single-strand breaks, which may subsequently lead to DNA double-strand breaks at replication forks [54]. In normal cells, double-strand breaks would be repaired in part by error-free homologous recombination DNA repair mechanisms [8]. Two proteins involved in this process are functional BRCA1 and BRCA2, which have a role in homologous recombination repair and maintenance of genomic stability [55]. If somatic mutations or epigenetic silencing leads to the absence of either BRCA1 or BRCA2, alternative DNA repair pathways such as nonhomologous end joining are employed; this subsequently results in chromosomal instability and cell death [54]. The use of PARP inhibitors in BRCA mutation carriers exploits the concept of synthetic lethality via combination of base excision repair inhibition with a defective homologous DNA repair pathway which results in the generation of unrepaired DNA single-strand breaks, an accumulation of double-strand breaks, collapsed replication forks, and eventual cell death [56–58]; see Figure 1.

Olaparib is an oral small-molecular PARP inhibitor. Preclinical studies confirmed that BRCA-deficient cells were up to 1000-fold more sensitive than wild-type cells to PARP inhibition [57]. Cells that are heterozygous for BRCA mutations, with an intact homologous recombination function, had a lack of sensitivity to PARP inhibitors similar to wild-type cells. This finding suggests that a therapeutic index for antitumor therapy may be present in BRCA-associated ovarian cancer [54, 57]. 

In phase I trials, olaparib was well tolerated, and there were no obvious differences in the pattern of toxicities between BRCA and non-BRCA patients [59–61]. A phase I trial in BRCA deficient ovarian cancer included 41 BRCA1 mutation carriers, 8 BRCA2 mutation carriers, and one patient with compelling family history for BRCA mutation. Of the 46 patients evaluable for RECIST or Gynecologic Cancer Intergroup CA125 response, 41% responded. An additional 11% of patients had meaningful stabilization of disease by RECIST criteria. Median response duration was 30 weeks. Responses were more frequent in the platinum-sensitive group, but were also seen in platinum-refractory and platinum-resistant populations. A phase I trial included 60 patients, 22 of which were carriers of a BRCA1 or BRCA2 mutation and one had a strong family history of BRCA-associated cancer but declined mutational testing [62]. The olaparib dose and schedule were increased from 10 mg daily for 2 of every 3 weeks to 600 mg twice daily continuously. Dose limiting toxicities including mood alteration, fatigue, thrombocytopenia, and somnolence seen at 400–600 mg twice daily led to the recruitment of a second cohort of patients consisting of only BRCA1 or 2 mutation carriers, who received olaparib at 200 mg twice daily. Objective antitumor activity was reported only in mutation carriers.

A randomized phase II trial comparing olaparib (200 or 400 mg orally twice daily) with pegylated liposomal doxorubicin (50 mg/m2 monthly intravenous) in patients with BRCA-mutated ovarian cancer with a platinum-free interval of 0–12 months is currently underway (NCT00628251). Another ongoing trial is a randomized placebo-controlled study of olaparib (400 mg orally twice daily) as maintenance therapy in patients with serous/sporadic ovarian cancer at high risk of early recurrence (NCT00753545).

## 9. Summary

Newer targeted therapies are undergoing evaluation in ovarian cancer. The most promising at this time are those directed towards inhibition of angiogenesis. Combining targeted therapeutics has resulted in significant toxicities, tempering enthusiasm for this approach. The finding of PARP inhibitors as potentially active agents in BRCA-associated ovarian cancer further supports the importance of screening patients for potential BRCA-associated disease and offering mutational testing when appropriate. Finally, given that the patient population who has typically entered trials evaluating targeted therapeutics includes those with recurrent or resistant disease, perhaps the finding of stable disease has some merit in the context of treatment effectiveness. Deeper understanding of biological pathways in ovarian cancer will be needed to select patients who enter these trials.

## Figures and Tables

**Table 1:** Results of three pivotal trials evaluating bevacizumab in ovarian cancer. Burger et al. and Cannistra et al. evaluated bevacizumab as a single agent whereas Garcia et al. evaluated bevacizumab with low-dose metronomic oral cyclophosphamide. All studies were performed in patients with recurrent disease.

Author	Progression free survival	Overall survival	Bowel perforation
Burger et al.	3.4 months	7.29 months	0%
Cannistra et al.	4.4 months	10.7 months	11.4%
Garcia et al.	7.2 months	16.9 months	5.7%

**Table 2:** Review of studies of antiangiogenic agents in recurrent or persistent ovarian cancer. RR: response rate, HTN: hypertension, RF: renal failure, P/S: platinum-sensitive, P/R: platinum resistant. *2 confirmed and one unconfirmed partial response. **1 unconfirmed partial response.

Agent	Authors	RR	Toxicities
VEGF trap	Tew et al.	5/45 partial	HTN, proteinuria, encephalopathy, RF
Cediranib	Hirte et al.	P/S: 3/17 partial*	Diarrhea, HTN, fatigue, anorexia
		P/R: 1/24 partial**	

**Table 3:** Review of drugs in the epidermal growth factor receptor family that have been evaluated in ovarian cancer. RR: response rate, SD: stable disease, HTN: hypertension.

Agent(s)	Authors	RR	SD	Toxicities
Gefitinib	Schilder et al.	1/27		Dermatologic, diarrhea
Gefitinib + tamoxifen	Wagner et al.	0/56	16/56	Diarrhea, skin rash
Erlotinib	Gordon et al.	2/34 partial	15/34	Rash, diarrhea
Erlotinib + bevacizumab	Nimeiri et al.	1/13 complete	7/13	Anemia, nausea, vomiting, HTN, diarrhea, 2 fatal GI perforations
		1/13 partial		
Transtuzumab	Bookman et al.	1/41 complete
		2/41 partial
Pertuzumab	Gordon et al.	5/107 partial	8/107	Diarrhea

**Table 4:** Review of multikinase inhibitors that have been studied in ovarian cancer. RR: response rate, SD: stable disease, HTN: hypertension, HFS: hand-foot syndrome.

Agent(s)	Authors	RR	SD	Toxicities
Sorafenib	Matei et al.	2/59 partial	20/59	Rash, GI, cardiovascular, metabolic, pulmonary
Sorafenib + bevacizumab	Azad et al.	6/13 partial		HTN, diarrhea, HFS, thrombocytopenia, proteinuria, fistula
Sorafenib + gemcitabine	Welch et al.	6/18 partial	10/18	Lymphopenia, thrombocytopenia, HTN, HFS, pain, neutropenia, hypokalemia
Imatinib	Coleman et al.	0/12	4/12	Fatigue, nausea/vomiting, rash, neutropenia
Imatinib	Alberts et al.	0/19		Hematologic, metabolic
Imatinib	Schilder et al.	1/56 complete		Neutropenia, GI, dermatologic, pain, electrolyte disturbances
